# Broadband magnetic resonance spectroscopy in MnSc$_2$S$_4$ 

**DOI:** 10.1038/s41598-023-37911-6

**Published:** 2023-07-08

**Authors:** Boglárka Tóth, Kirill Amelin, Toomas Rõõm, Urmas Nagel, Anastasia Bauernfeind, Vladimir Tsurkan, Lilian Prodan, Hans-Albrecht Krug von Nidda, Marc Scheffler, István Kézsmárki, Sándor Bordács

**Affiliations:** 1 Department of Physics, Institute of Physics, Budapest University of Technology and Economics, Műegyetem rkp. 3., H-1111 Budapest, Hungary; 2 National Institute of Chemical Physics and Biophysics, Akadeemia tee 23, 12618 Tallinn, Estonia; 3 Experimental Physics V, Center for Electronic Correlations and Magnetism, Institute of Physics, University of Augsburg, 86159 Augsburg, Germany; 4 Institute of Applied Physics, Moldova State University, 5 Academiei Str., 2028 Chisinau, Republic of Moldova; 5 1. Physikalisches Institut, University of Stuttgart, Pfaffenwaldring 57, 70569 Stuttgart, Germany; 6 ELKH-BME Condensed Matter Research Group, Budapest University of Technology and Economics, Műegyetem rkp. 3., H-1111 Budapest, Hungary

**Keywords:** Magnetic properties and materials, Topological matter

## Abstract

Recent neutron scattering experiments suggested that frustrated magnetic interactions give rise to antiferromagnetic spiral and fractional skyrmion lattice phases in MnSc$_2$S$_4$ . Here, to trace the signatures of these modulated phases, we studied the spin excitations of MnSc$_2$S$_4$ by THz spectroscopy at 300 mK and in magnetic fields up to 12 T and by broadband microwave spectroscopy at various temperatures up to 50 GHz. We found a single magnetic resonance with frequency linearly increasing in field. The small deviation of the Mn$^{2+}$ ion *g*-factor from 2, *g* = 1.96, and the absence of other resonances imply very weak anisotropies and negligible contribution of higher harmonics to the spiral state. The significant difference between the dc magnetic susceptibility and the lowest-frequency ac susceptibility in our experiment implies the existence of mode(s) outside of the measured frequency windows. The combination of THz and microwave experiments suggests a spin gap opening below the ordering temperature between 50 GHz and 100 GHz.

## Introduction

There has been a continued interest in materials with competing magnetic interactions as they may give rise to highly correlated fluctuating states^1,2^ or exotic magnetic orders^3–6^. To minimize the magnetic energy, as a compromise, spin spirals described by a single $\textbf{q}$-vector often emerge in these compounds with frustrated interactions^7–12^. Recent theoretical works on frustrated magnets indicated that quartic-order terms in the Landau free energy can even stabilize a rich variety of multi-$\textbf{q}$ spin states, including topologically non-trivial magnetic skyrmions^13–15^. The helicity and vorticity of skyrmions can fluctuate, allowing the manipulation of these internal degrees of freedom, which may couple to electric polarization^14,15^. Very recently, nanoscale skyrmions were detected in centrosymmetric Gd magnets with triangular^16^, kagome^17^ and square lattices^18^, initializing experimental studies of magnetic skyrmions in frustrated magnets.

The bipartite diamond lattice can also become frustrated with competing nearest ($J_1$) and next-nearest neighbor ($J_2$) interactions^19,20^. MnSc$_2$S$_4$ realizes this exchange-frustrated model, as the magnetic ${\rm Mn}^{2+}$ ions with $S=$ 5/2 spins occupy the diamond sublattice of its spinel structure^21^. The Curie-Weiss temperature $\Theta _{CW}=-$22.9 K is an order of magnitude larger than the magnetic ordering temperature $T_N=$ 2.3 K, implying that the magnetic interactions are strongly frustrated in this compound^21,22^. According to neutron scattering experiments, above the ordering temperature $T_N$, the fluctuations are correlated and a unique spiral spin liquid state emerges^22^. This state orders into a sinusoidally modulated collinear phase at $T_N$, which becomes incommensurate below 1.64 K and, finally, transforms to a helical state below 1.46 K. Furthermore, elastic and inelastic neutron scattering experiments suggested that in a finite magnetic-field, a triple-$\textbf{q}$ state is stabilized, which, based on Monte Carlo simulations, is associated with a fractional antiferromagnetic skyrmion lattice^23^.

The spectroscopy of the magnetic resonances has been proven to provide valuable information on the magnetic order and allows accurate determination of microscopic interaction parameters^24^. Since the spin spiral has a periodicity larger than that of the chemical unit cell, there is a folding of the spin-wave dispersion into the smaller Brillouin zone of the spin spiral, a series of excitations may emerge in the $\Gamma$-point ($k=0$), that can be probed by absorption spectroscopy with high energy resolutions^25–28^. Such modes corresponding to the distortion of the phase or collective tilt of the plane of the spiral have been found, for example, in orthorhombic manganites^29^, $\hbox {BiFeO}_3$^30–32^, cubic chiral helimagnets^33,34^, and in $\hbox {Cu}_2\hbox {OSeO}_3$^35^. The higher dimensional multi-$\textbf{q}$ states may give rise to additional modes as in the case of skyrmion lattice, where a breathing, a clockwise and a counterclockwise rotational mode were predicted and observed^34–36^. Very recent analytical calculations and numerical simulations show that an antiferromagnetic skyrmion lattice stabilized in synthetic antiferromagnet also possesses a phason mode and a series of optical magnons^37^.

The aim of this study is to investigate the magnetic-field dependence of the spin excitations in MnSc$_2$S$_4$ by THz spectroscopy up to 17 T. We carried out the experiments in the paramagnetic phase at 2.5 K, as well as in the ordered state at 300 mK, where the zero-field ground state is the helical spiral state and in the 4.5–7 T field range the triple-$\textbf{q}$ state is expected to emerge^22^. We performed additional GHz experiments at 300 mK and from 2 K up to 20 K to address lower frequency and field ranges.

## Results

### THz absorption

We measured light absorption of a MnSc$_2$S$_4$ mosaic in the far-infrared range, between 100 GHz and 3 THz at 2.5 K, and between 100 GHz and 2.1 THz at 300 mK.

**Figure 1:**
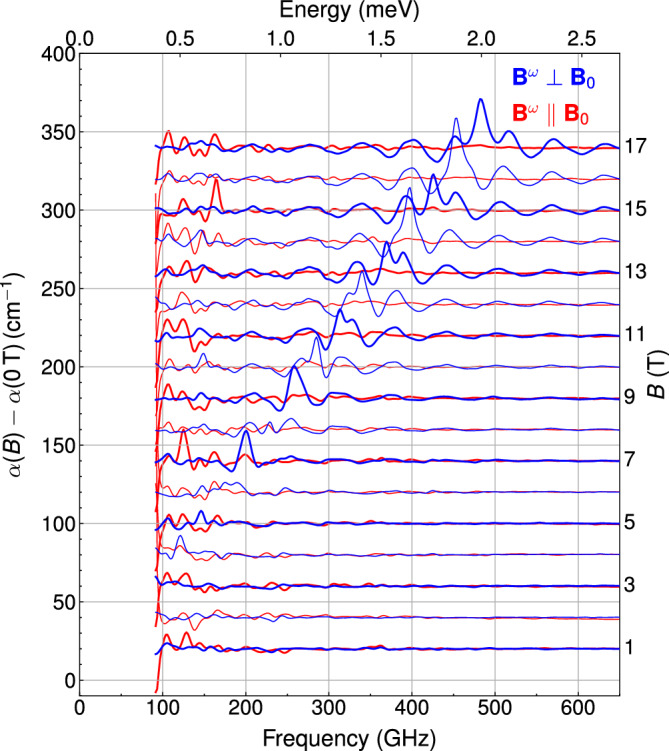
Magnetic field and polarization dependent THz absorption spectra measured in Voigt configuration in MnSc$_2$S$_4$ at $T=$ 2.5 K. The absorption differences are shown with respect to zero field spectrum in magnetic fields up to 17 T. Bold spectra were measured at odd field values. Undulation of the spectra in the vicinity of the resonance is due to multiple reflections within the plane-parallel sample that is distorted by the change of sample optical constants near the spin resonance mode.

Figure 1 shows the field dependence of the differential absorption spectra at 2.5 K. We resolved a single paramagnetic resonance, which shifts linearly with field. The wavy baseline in the vicinity of the peak is caused by the magnetic-field-induced change of the interference pattern, arising due to multiple reflections in the nearly plane-parallel sample. A linear fit on the magnetic field dependence of the resonance frequency results in a 27 ± 0.6 GHz/T slope, and a zero-field offset of 20.4 ± 7.2 GHz. The slope corresponds to a *g*-factor of 1.93 ± 0.05. The fact that we did not detect any deviation from the linear field dependence of the resonance apart from a small off-set suggests that there is a small anisotropy of spin Hamiltonian parameters. The small anisotropy is also consistent with the small deviation of *g*-factor from the free electron value, both caused by the spin-orbit coupling. The resonance line appears only when the direction of the alternating magnetic field is perpendicular to the external magnetic field, $\textbf{B}^{\omega }\perp \textbf{B}_{0}$, and is absent when $\textbf{B}^{\omega }\parallel \textbf{B}_{0}$, which is consistent with a simple paramagnetic behaviour.

**Figure 2:**
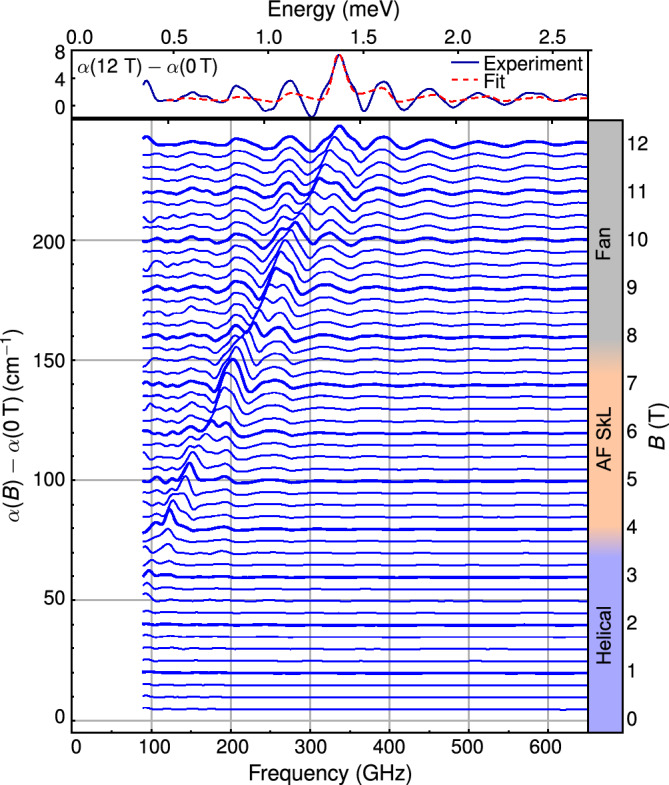
THz absorption spectra in Faraday configuration for unpolarized radiation at *T* = 300 mK. In the bottom panel, the magnetic field dependence of the THz absorption spectrum is shown. Spectra measured in integer fields are plotted with thick lines. Top panel shows the spectrum measured in 12 T (blue line) and its fit to Lorentzian model (red dashed line). Side bar shows the field regions where the magnetic phase has helical, antiferromagnetic skyrmion lattice (AF SkL), and fan structure according to Ref. ^23^.

Figure 2 shows the field dependence of the absorption spectra relative to zero field at 300 mK. The spectra follow a similar magnetic-field dependence as the ones measured at 2.5 K. The linear fit to the resonance peak positions shows a resonance shift of 27.5 ± 0.4 GHz/T, and a zero-field offset of 9.5 ± 1.5 GHz. The calculated slope corresponds to a *g*-factor of 1.96 ± 0.02, which is the same as that of deduced in the paramagnetic phase within the error of the measurement. The finite frequency intercept is somewhat smaller as compared to the paramagnetic resonance at 2.5 K.

Neither in the ordered nor in the paramagnetic phase could we resolve clear deviation from the linear field dependence. If there is any anisotropy induced gap, it is below 100 GHz, the low-frequency cut-off of our experiment. The spin resonance detected at 300 mK is not sensitive to the magnetic phase transitions that are suggested to occur at 4.5 T and 7.5 T, according to Ref. ^23^. Moreover, in our frequency window, we did not detect any other resonances, which may arise due to the emergence of a modulated spin structure, such as a spin spiral or magnetic skyrmion lattice. The absence of any signature of the modulated phase might correspond to the weak spin-orbit interaction and the related weak magnetic anisotropy of $\hbox {Mn}^{2+}$. Without a sizable magnetic anisotropy, the spin spiral is harmonic and the modes folded to the reduced Brillouin zone remain silent. The oscillation of the plane of the harmonic spiral may induce modulation of the electric polarization via the inverse Dzyaloshinskii-Moriya coupling^26,27^, however, this mechanism is active only for spin cycloids, i.e., it cannot generate infrared active modes in the helical state of MnSc$_2$S$_4$ . These are the most likely reasons for not observing additional spin resonances in the covered spectral range.

From the absorption spectrum, the $\omega$ $\rightarrow$ 0 magnetic susceptibility $\chi$ can be obtained using the Kramers-Kronig relations, assuming that $\chi$ is small and the dielectric function $\varepsilon$ is constant in the THz range^38^:

1 $$\begin{aligned} \chi (\omega \rightarrow 0) = \frac{2}{\pi }\frac{c}{\sqrt{\varepsilon }}\int _{0}^{\infty } \frac{\alpha (\omega )}{\omega ^{2}} {\textrm{d}}\omega , \end{aligned}$$

where $\alpha$ is the absorption coefficient, $\omega$ is the angular frequency, and *c* is the speed of light in vacuum. We fitted the experimental $\alpha (B)-\alpha (0\,{\textrm{T}})$ spectrum at *B* = 12 T with a single resonance, see top panel of Fig. 2 for illustration. The magnetic susceptibility is described by a Lorentzian oscillator:

2 $$\begin{aligned} \chi (\omega ) = \frac{S}{\omega _0^2-\omega ^2-i\omega \gamma } \end{aligned}$$

where $\omega _0$ is the resonance frequency, $\gamma$ is the damping parameter and *S* is the oscillator strength. To take into account multiple reflections within the sample, we modeled it as a Fabry-Perot etalon with infinite number of reflections. By assuming that the resonance is absent in zero field, our model provided an estimate for the THz dielectric constant: $\varepsilon$ = 12.1. The evaluation of the integral in Eq. (1) with the Lorentzian model gave $\chi (\omega \rightarrow 0)$ = 2.5$\times$10$^{-3}$ for fields oscillating perpendicular to the static field. This transverse susceptibility is an order of magnitude smaller than $\chi _0$ = 0.021, the value published for the longitudinal, static susceptibility in Ref. ^21^. Since the magnetization curve is nearly linear even in the magnetically ordered phases^23^, and the anisotropy is weak, these transverse and longitudinal susceptibilities should be nearly equal in the static limit. The missing spectral weight, i.e., the difference between $\chi (\omega \rightarrow 0)$ and $\chi _0$, must lie outside of the frequency range of our measurement system, implying the presence of further resonance(s) below 100 GHz, the low-frequency cutoff of the present study. In fact, an antiferromagnetic spiral emerging due to exchange frustration has three Goldstone modes in the absence of anisotropy: a phason mode, $\Phi _0$ corresponding to rotations within the plane of the spiral and two others, $\Psi _{\pm 1}$ associated to out-of-plane rotations^28^. Magnetic anisotropy terms compatible with cubic symmetry may gap these modes making them detectable with microwave spectroscopy. Finally, we mention that non-resonant, relaxation modes may also explain the missing spectral weight as inferred in the case of the frustrated magnet $\hbox {ZnCr}_2$O$_4$^39^.

**Figure 3:**
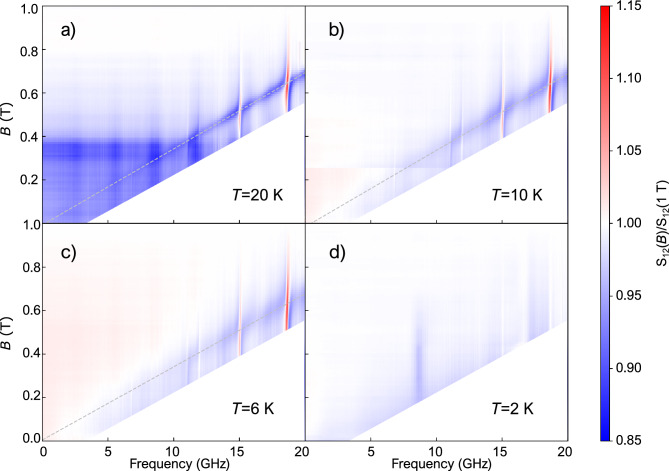
(**a–c**) Low frequency broadband transmission ratios in the paramagnetic temperature range. Dashed lines indicate a linear fit to the resonance positions. (**d**) Low frequency broadband transmission ratios below the magnetic ordering temperature.

### Microwave transmission

In order to search for lower frequency excitations, we also performed broadband microwave transmission measurements in the paramagnetic phase as well as in the magnetically ordered modulated phases. In the paramagnetic phase *T* > $T_N$, our measurements covered the 10 MHz to 20 GHz and 0–1 T frequency-magnetic field range as shown in Fig. 3. We observed a single resonance from the sample that is shifted linearly with the field. The *g*-factor of this line without any zero-field offset at *T* = 20 K, *T* = 10 K, and *T* = 6 K is 2.07, 2.08, and 2.1, respectively, for fields applied along $\mathbf {\textrm{B}}\parallel$ [111]. The linewidth became broader as the temperature approached the Néel temperature and the resonance was not visible at 2 K. Although we extended our experiments up to 50 GHz and 8 T in the magnetically ordered phase, we did not detect any resonance from the sample in the 300–600 mK temperature range. The combination of the microwave and THz results suggests that the spin resonance of MnSc$_2$S$_4$ occurs in the 50–100 GHz frequency window in zero field, i.e. a spin gap is opening below $T_N$.

## Conclusions

Earlier elastic and inelastic neutron scattering studies combined with Monte Carlo simulations found multiple phases in MnSc$_2$S$_4$ , including a multi-$\textbf{q}$ state, such as an antiferromagnetic skyrmion phase. Motivated by these findings, we studied the magnetic field dependence of the spin excitations in MnSc$_2$S$_4$ by THz and microwave spectroscopy in the paramagnetic phase as well as in the ordered state. Although the material has a rich phase diagram with multiple modulated magnetic phases, we only observed a single resonance, whose frequency does not exhibit anomalies at the critical fields separating these phases. This resonance has *g*-factor close to 2 and shows no deviation from the linear field dependence in the studied frequency range, which indicates a small anisotropy. Other collective modes of the modulated states were not detected likely due to their negligible magnetic dipole activity, being the consequence of weak magnetic anisotropy. The analysis of the intensity suggests that further spin excitation(s) should be present outside of the measured frequency-magnetic field windows.

## Methods

Single crystals with a typical size of $\sim$1 $\hbox {mm}^3$ were grown by chemical transport technique, as described in Ref.^22^. Several co-oriented crystals facing to the [111] direction were glued to obtain a mosaic with $\sim$2 mm diameter and 0.65 mm thickness. Sub-Kelvin temperatures were reached in a modified Oxford TLE200 wet dilution refrigerator at the National Institute of Chemical Physics and Biophysics (KBFI), Tallinn. The propagation vector of the incident unpolarized light was parallel to the external magnetic field, which is the so-called Faraday configuration. Measurements at 2.5 K were also performed at KBFI, on the TeslaFIR cryostat setup. These measurements were performed with polarized light, in Voigt configuration, i.e. propagation vector of the exciting polarized light was perpendicular to the external magnetic field. In both cases, the spectra were measured with an SPS200 far-infrared Martin-Puplett interferometer and a 300 mK silicon bolometer.

The field-induced change in the absorption coefficient $\alpha$ was calculated as

3 $$\begin{aligned} \alpha (B) - \alpha (0\,{\textrm{T}})= -\frac{1}{d}{\textrm{ln}}\left( \frac{\mathscr {I}(B)}{\mathscr {I}(0\,{\textrm{T}})}\right) , \end{aligned}$$

where $\mathscr {I}(B)$ is transmitted light intensity at a specific magnetic field *B*, and *d* is the sample thickness.

Low frequency broadband measurements were performed in the microwave laboratory at Universität Stuttgart using metallic coplanar waveguides (CPWs)^40^. Measurements at and above 2 K were done with a 20 GHz vector network analyzer (VNA) in a magnet cryostat with variable temperature insert. The frequency-magnetic field maps were recorded as frequency sweeps in constant magnetic field. Measurements in the sub-Kelvin temperature range were performed with a 50 GHz VNA, in a wet dilution refrigerator on a single crystal with typical sizes 2.46 mm $\times$ 2.11 mm.

## Data Availability

The datasets analysed during the current study are available from the corresponding author on reasonable request.
